# Nordcan.R: a new tool for federated analysis and quality assurance of cancer registry data

**DOI:** 10.3389/fonc.2023.1098342

**Published:** 2023-08-08

**Authors:** Siri Larønningen, Anna Skog, Gerda Engholm, Jacques Ferlay, Tom Børge Johannesen, Marnar Fríðheim Kristiansen, Daan Knoors, Simon Mathis Kønig, Elinborg J. Olafsdottir, Sasha Pejicic, David Pettersson, Charlotte Wessel Skovlund, Hans H. Storm, Huidong Tian, Bjarte Aagnes, Joonas Miettinen

**Affiliations:** ^1^ Department of Registration, Cancer Registry of Norway, Oslo, Norway; ^2^ Cancer Surveillance and Pharmacoepidemiology, Danish Cancer Society Research Center, Copenhagen, Denmark; ^3^ Cancer Surveillance Branch, International Agency for Research on Cancer, Lyon, France; ^4^ University of the Faroe Islands, Faculty of Health Sciences, Tórshavn, Faroe Islands; ^5^ Research and Development, Netherlands Comprehensive Cancer Organisation, Eindhoven, Netherlands; ^6^ The Icelandic Cancer Registry, Reykjavik, Iceland; ^7^ The National Board of Health and Welfare, Stockholm, Sweden; ^8^ Finnish Cancer Registry, Helsinki, Finland

**Keywords:** NORDCAN, quality, harmonization, cancer, epidemiology, software, GDPR-compliance, federated analysis

## Abstract

**Aim of the article:**

We present our new GDPR-compliant federated analysis programme (nordcan.R), how it is used to compute statistics for the Nordic cancer statistics web platform NORDCAN, and demonstrate that it works also with non-Nordic data.

**Materials and methods:**

We chose R and Stata programming languages for writing nordcan.R. Additionally, the internationally used CRG Tools programme by International Agency for Research on Cancer (IARC/WHO) was employed. A formal assessment of (GDPR-compliant) anonymity of all nordcan.R outputs was performed. In order to demonstrate that nordcan.R also works with non-Nordic data, we used data from the Netherlands Cancer Registry.

**Results:**

nordcan.R, publicly available on Github, takes as input cancer and general population data and produces tables of statistics. Each NORDCAN participant runs nordcan.R locally and delivers its results to IARC for publication. According to our anonymity assessment the data can be shared with international organizations, including IARC. nordcan.R incidence results on Norwegian and Dutch data are highly similar to those produced by two other independent methods.

**Conclusion:**

nordcan.R produces accurate cancer statistics where all personal and sensitive data are kept within each cancer registry. In the age of strict data protection policies, we have shown that international collaboration in cancer registry research and statistics reporting is achievable with the federated analysis approach. Undertakings similar to NORDCAN should consider using nordcan.R.

## 1 Introduction

The Nordic cancer registries have collaborated closely since the first registries were established in the 1940s and early 1950s. The collaboration was formalized in 1966 as the Association of Nordic Cancer Registries (ANCR) and has resulted in numerous projects and collaborations to describe incidence and mortality trends in the Nordic countries and to develop statistics to support cancer surveillance, decision making and research in the Nordic countries (1–7). A crucial part of these projects is to prove the quality of the Nordic cancer registry data through quality estimates like completeness, validity, timeliness and comparability (8, 9).

NORDCAN, the Nordic cancer database and webtool for cancer incidence, mortality, prevalence and survival (10) was created in the mid-90’s in a collaboration between the International Agency for Research on Cancer (IARC) and driving forces in some of the Nordic cancer registries. NORDCAN on the web was established in 2002. A work group including representatives from the cancer registries in all the Nordic countries was established to maintain and develop NORDCAN. The history, organization and use of NORDCAN and the comparability of the Nordic cancer registries have been described earlier (11–13).

**Figure 1:**
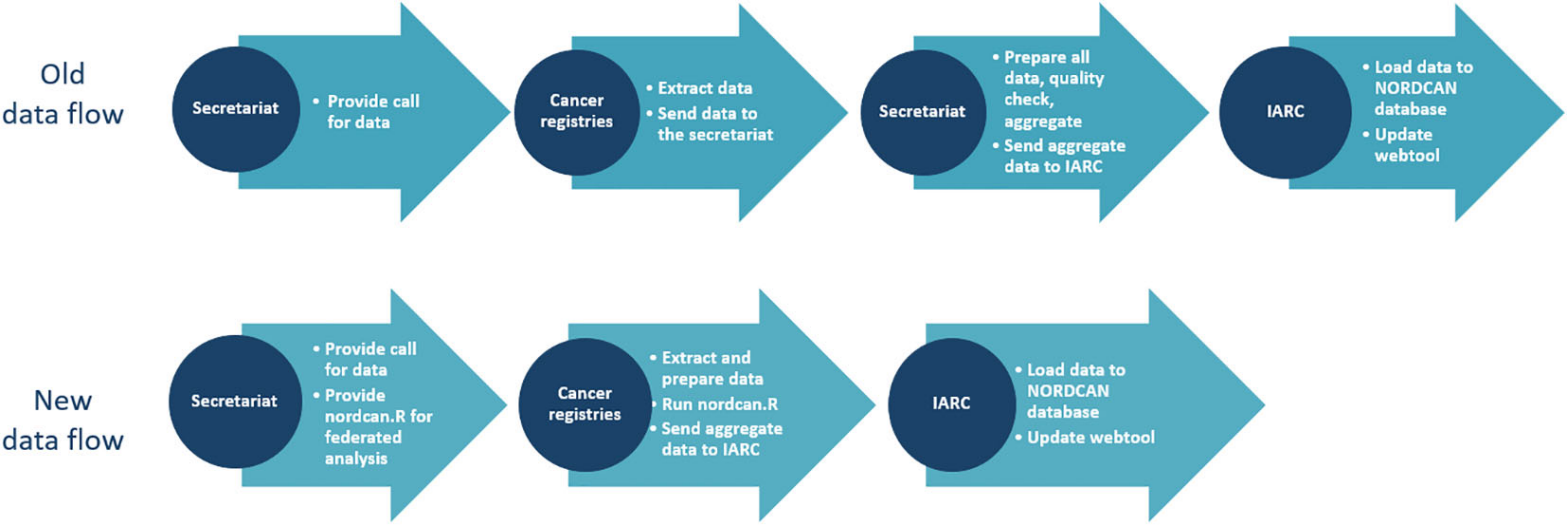
Old and new data flow for NORDCAN.

The European General Data Protection Regulation (GDPR) (14) in effect since 2018, strengthened standards for sharing personal data. Although the GDPR allows for sharing personal data within the European Union and EEA, the stricter statutory interpretation of GDPR in most Nordic countries creates additional barriers. This includes the definition of personal data, individual consent to research use and international collaboration. Sharing personal data with UN agencies such as the IARC (WHO), which are exempted from national legislation and thus not subject to the GDPR regulations, is not allowed. It became apparent that the established NORDCAN procedures on data extraction, data preparation and data processing should be changed. The old and new data flow are shown in **Figure 1**.

The counts and statistics produced by nordcan.R are sent to IARC through a file transfer and are stored in the NORDCAN database to be visually represented in the NORDCAN webtool. Incorporated in the webtool is the logic to perform calculation of rates, predictions, estimated annual percentage change and survival improvement, based on the output files from nordcan.R. All statistics are based on the counts and analyses done in nordcan.R, and rates are available using the standard populations World, Europe and Nordic.

The aim of this article is to present the new GDPR compliant federated analysis programme (nordcan.R), how it is used to compute statistics for NORDCAN and how it can be used also for non-Nordic data.

## 2 Materials and methods

In 2018 we started rebuilding the data management, quality control, data flow and webtool of NORDCAN on research grants from the Danish Cancer Society, the Nordic Cancer Union (NCU) and in kind-contributions from the Nordic cancer registries.

There were two key issues to address. First, we had to ensure that personal data on cancer patients, in line with the national implementation of GDPR, would not be released from the cancer registries. Second, we had to make sure that released (aggregated) data to the NORDCAN database in IARC were comparable between countries and to previous releases.

To keep personal data on cancer patients within each cancer registry, we developed a simple federated system; a program that could be run separately in each country, producing all counts and statistics necessary for NORDCAN. The program was named nordcan.R. We established an IT-group with representatives from each of the Nordic cancer registries. The IT-group decided on architecture, technology, software and development guidelines to be used for the federated analysis application, developed the application itself, and tested the tool. Prior experience in designing and programming a system for data preprocessing and aggregation for the Finnish Cancer Registry was taken as the model.

We decided to use R (15) and Stata (16) for the development of the new tool. In addition, we chose to continue using the IARC check and conversion program (17) for conversions from International Classification of Diseases for Oncology, third edition (ICD-O-3) (18) to International Classification of Diseases, tenth edition (ICD-10) (19) and for implementation of the multiple primary rules (20).

We specified four common input datasets to be used in nordcan.R: a cancer case dataset (21), a cancer death dataset (22), population datasets (including population projections) (23) and life tables to be used in survival analysis (24). Specifications on cancer entities (the cancer dictionary) used in NORDCAN already existed, but were revised to better meet the current needs of clinicians, cancer unions, patient organizations, researchers, and politicians (25, 26). We also developed a conversion table for mortality to be used for the conversion of old ICD-codes (ICD6-9) to ICD-10 (27).

Before starting the development of nordcan.R, we identified the core functionality of the program (**Table 1**). The NORDCAN secretariat also provided all participating countries with an overview of the necessary tools to be used, with links to installation files and sources, and additional information on licensing where necessary (**Table 2**).

**Table 1:** Identified core functionality of nordcan.R.

Core functionality	Specification
**User-specified global settings**	The user should be allowed to set: - Country - First and last year of national incidence data - First and last year of national mortality data - Last year of follow-up for survival data - First year of regional data
**Check of input files**	nordcan.R should check that the input-files adhere to the call for data
**Integration with the IARC check and conversion program**	nordcan.R should create the necessary input files for the IARC check and conversion program, and be able to read the output files from this program back into nordcan.R.
**Enrichment of original data**	nordcan.R should enrich the original data using output-files from the IARC check and conversion program and specifications from the NORDCAN group. This includes grouping data into NORDCAN entities and documenting reasons for exclusion of cases. nordcan.R should do recoding and calculations necessary for the final computation of data.
**Create necessary output files**	nordcan.R should create the following output: A zip-file with all counts and analyses necessary for the NORDCAN database - Counts for cancer cases, cancer deaths and prevalent cases by year, sex, region/country and entity (28) - Age standardized relative survival using the Pohar Perme estimator via stnet in Stata (29, 30). A folder with maintainer summary files - Technical information - Comparison summary - Graphics comparing counts between two different runs

**Table 2:** Tools necessary for nordcan.R users.

Tool	
**R**	https://www.r-project.org/ We recommend using R-studio (https://rstudio.com), but it is not necessary to run nordcan.R.
**Stata** (Stata/IC is sufficient)	https://www.stata.com/
**IARCcrgTools**	http://www.iacr.com.fr/index.php?option=com_content&view=article&id=72:iarccrgtools&catid=68&Itemid=445

We did a risk assessment on the aggregate data of the output files (31). We drew knowledge from other sources on statistical disclosure control and data anonymization (32–34) to evaluate the risk of disclosure from the datasets created by nordcan.R and to identify possible risk-reducing measures.

We established a proof-of-concept project between the NORDCAN secretariat and the Netherlands Comprehensive Cancer Organization (IKNL) to test the usability of nordcan.R outside of the Nordic countries. We used data from the Netherlands Cancer Registry (NCR) hosted by IKNL for the period 2000-2019. For the comparison of the output, we calculated all rates in Stata, using the World standard population (35) for age-adjusted rates.

## 3 Results

The first version of nordcan.R (nordcan_9.0_1.0) was published to the NORDCAN-participants on November 27^th^, 2020. The most recent, nordcan_9.3_1.3, was released May 4^th^, 2023. The application has so far been used to update data in NORDCAN from 2016 to 2021.

The main result of revamping the software was that all Nordic countries were able to deliver updated, anonymous, tabular data to the NORDCAN database and webtool, hosted by IARC, without being subject to legal discussions and hindrance due to differences in the interpretation of GDPR. Through nordcan.R, all data were quality assured, converted, analyzed and counted the same way, and the same inclusions and exclusions were applied to all data, regardless of country and region.

**Figure 2:**
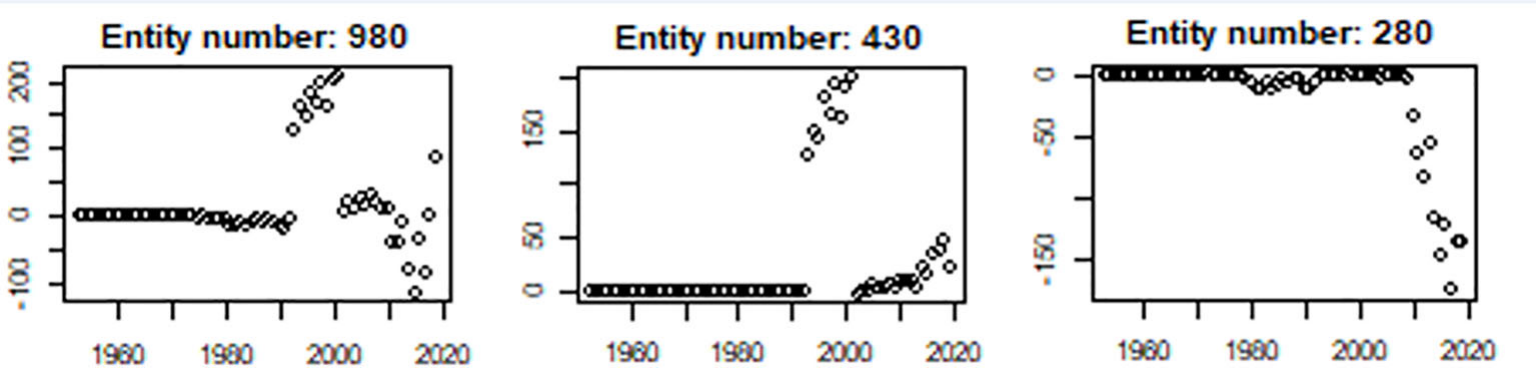
Comparison between previous and current number of incident cases for selected entities produced as a standard output by nordcan.R. The Y-axis represents change in number of cases, the X-axis represents year of cancer counts. The changes in all sites (980) are mainly driven by changes in malignant hematopoietic diseases (430) and urinary tract cancers (280).

Nordcan.R produces a “maintainer summary” with plots and tables comparing the newly created counts to those created in a previous call for data. The user is required to inspect these comparisons for unexpected discrepancies between two versions as an additional quality assurance step. **Figure 2** shows a comparison of counts of cancer cases in Norway between version 9.1 and version 9.2 of NORDCAN for selected entities. The reasons for changes in entity 980 (all sites) is clearer when looking at visualizations for each specific entity. We see an increase in entity 430 (malignant hematopoietic diseases), and changes in entity 280 (urinary tract cancers). Both these changes are explained by a thorough process of quality assurance and corrections in the Cancer registry of Norway. Smaller changes to the cancer counts, mainly caused by regular day-to-day corrections in cancer registries, are expected. The maintainer summary serves to acknowledge that each nordcan.R-user has seen the results for their own country and the comparison with previous results and accepts the results as valid. The maintainer summary is also an indication that the technical requirements of nordcan.R have been fulfilled.

**Figure 3:**
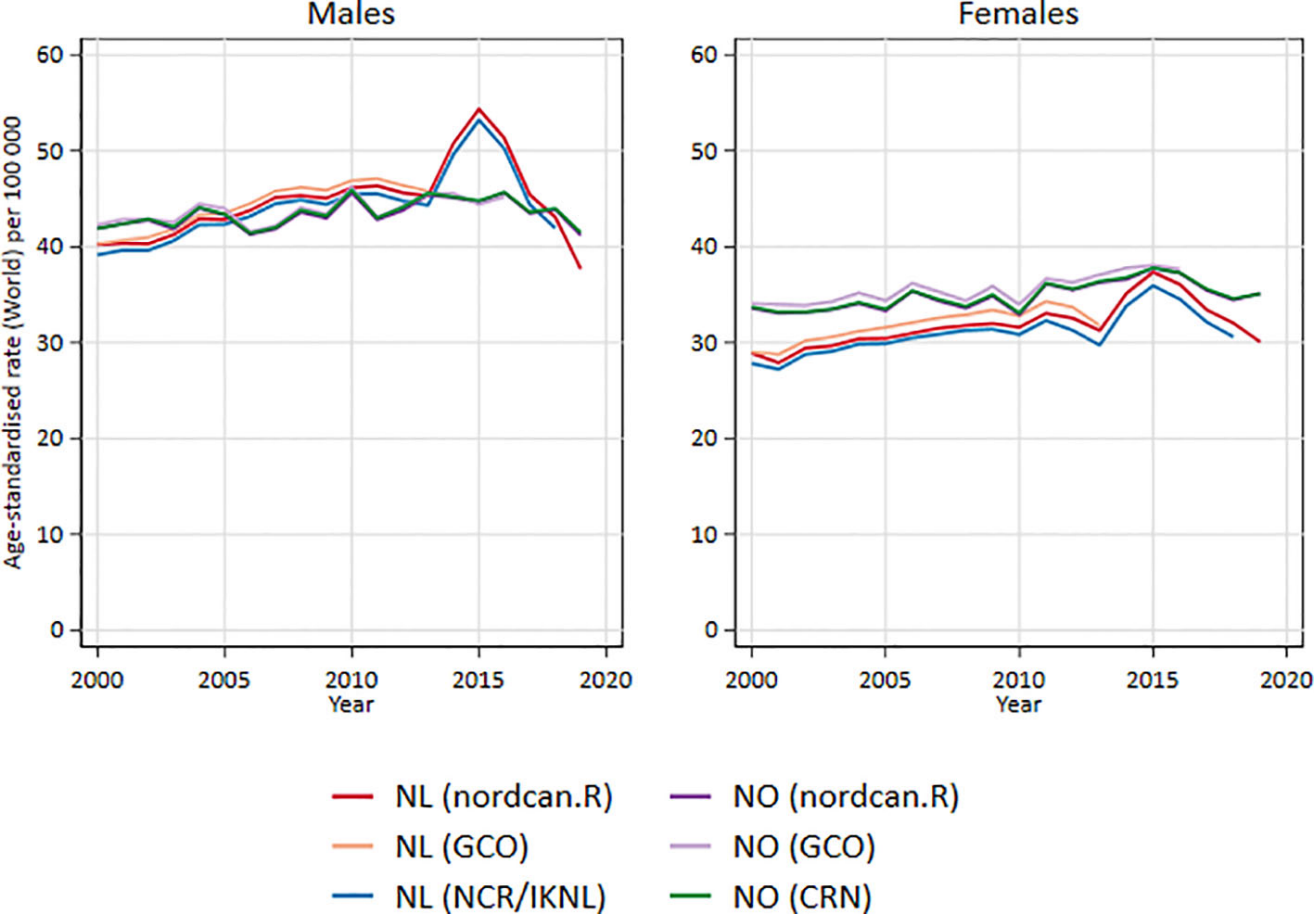
Comparison of adjusted incidence rates (World) between GCO, official national statistics and nordcan.R for colorectal cancer in Netherland and Norway, men and women separately.

**Figure 3** shows the comparisons of adjusted incidence rates (World) between The Global Cancer Observatory (GCO) (36), official cancer statistics in the Netherlands (37) and Norway (38) and results from nordcan.R for colorectal cancer, men and women shown separately. There are only minor differences.

## 4 Discussion

As shown in the results, we achieved our main goal. We have produced the necessary data and we have been able to share the data with IARC for updating the NORDCAN database and webtool.

As GCO and nordcan.R both use the IARCcrgTool for conversions and exclusions of multiple primary cancers, and both cancer registries adhere to international rules on cancer registration, we expected that the trend lines would be quite similar. It is still valuable to make this comparison to see that nordcan.R did not introduce any unforeseen errors. Comparison with official national statistics produced by the national cancer registries is of additional value to see that the numbers and rates in NORDCAN do not deviate even if the IARCcrgTool is not used. The slight differences we see might be due to changes in the original data caused by updates, differences in implementation of multiple primary rules or corrections or small differences in exclusions and inclusions of cases.

We have a common call for data to be adhered to by all participants. We aimed to make this call for data as similar as possible to calls for data issued by IARC for Cancer Incidence in Five Continents (CI5) – Volume XII (39) and by The European Network of Cancer Registries (ENCR) in collaboration with the Joint Research Centre of the European Commission (JRC) for the update of the European Cancer Information System (ECIS) (40). NORDCAN has, however, focused mainly on the traditional cancer registry-variables which are needed to calculate incidence, mortality, prevalence and survival on country and regional level, whereas the JRC/ENCR call for data also includes variables on stage, treatment and more detailed geographical area.

Incorporating quality checks, conversions and inclusions/exclusions into the data management process when running nordcan.R, and using transparent and unified specifications were discussed in the NORDCAN work group as important steps towards comparability. This is a well-known recipe, used by both IARC and ENCR-JRC, and was also the same process used previously in NORDCAN. In the current NORDCAN pipeline, each country is responsible for completing the entire process themselves, whereas in the old, centralized approach, each participant sent data on patients to one central node for checks, conversions and aggregation. A big advantage of the new procedure is that the cancer registries run their own data through the entire process and see all errors and warnings. Another big advantage – and a prerequisite for the development of nordcan.R – is that all personal and sensitive health data is kept within each cancer registry. The disadvantage of this data flow is that there is a possibility that the countries select and map their original data in a slightly different way to fit the call for data, and thereby introduce a few minor differences. However, taking all pros and cons into consideration, our view is that the cost of introducing a few differences has less impact than the benefit of users with first-hand knowledge of the data doing all data processing, aided by a tailor-made tool.

Using a common check and conversion program like the one from IARC is also an advantage, as it ensures that all data in NORDCAN have been subject to the same conversion and check rules. A disadvantage of the current version of the IARC check and conversion program is that it is not updated with the most recent morphology codes from the ICD-O-3 second revision. Also, although nordcan.R includes written instructions on what selections to use in the tool, there is still room for manual errors that can lead to data being less comparable.

In the current version of nordcan.R, all entities or cancer groups are predefined through a table in the application. The user cannot change the groups or run different specifications without reprogramming parts of nordcan.R. This is an advantage, as it ensures comparability as to which ICD10-codes are included in the different entities, and which morphologies might be included or excluded in addition to the ICD10-codes, but makes nordcan.R less useful if the user wishes to prepare statistics for other cancer sites or wishes to compare to international statistics with different cancer groups.

When running nordcan.R, the user gets a visualization and an output showing how similar the current counts are to previous counts. Although this does not ensure comparability between countries, it ensures that data within one country are comparable over time, and that any bigger changes affecting the comparability of data can be documented and explained. Most cancer registries are “living organisms”, where data are changed and updated over time, also for previously published data. It is therefore not unusual to see changes in counts from one version of NORDCAN to the next, but the reasons for changes should be clear and well documented, and any bigger changes should be accompanied by a warning on the webpage.

The usability of nordcan.R outside of NORDCAN was tested in our proof-of-concept project with Dutch cancer data. The first main hindrance was the necessity of installing three different types of software to run nordcan.R: R and the R-packages, Stata, and the IARC check and conversion tool. As nordcan.R operates on sensitive data, all tools needed to be installed in a secure environment which allowed for this kind of data processing. For Stata, there is an additional hindrance that installation and use require a license which the institution might not be willing to purchase. Not every potential user can fulfill the requirement of an on-site Stata installation. However, this only excludes the computation of survival statistics while other statistics are unaffected. Preparing a dataset according to the specifications given for nordcan.R did not reveal any major problems for IKNL. An upcoming inclusion of quality tables will also make it easier to compare reasons for exclusions and other quality measures of the data (41).

### 4.1 Future perspectives

The steps taken so far are only an introduction to future challenges and possibilities. Although nordcan.R fulfills its purpose for the current requirements of NORDCAN, there are still missing parts. For instance: we can currently show survival in Norway and Sweden compared to each other, but not survival for Norway and Sweden together.

With the current problems we have in sharing data with IARC, we see the need for a better alternative. This is why we look to federated analysis as one possible solution for sharing data and knowledge across borders without the current hindrances we meet in laws and regulations. A future incarnation of NORDCAN should allow for more complex analyses than are currently possible and retain the successful federated analysis approach. Establishing common data models in each cancer registry, for instance using the OMOP Common Data Model (42), might make it easier to implement different federated learning infrastructures to run analysis on local data and only share the results. However, getting approval to install software on-site which enables external access to sensitive data is not straightforward.

## 5 Conclusion

The Nordic cancer registries successfully developed a new software tool (nordcan.R) which enables them to share aggregated cancer statistics, comparable to previous releases and in a GDPR compliant manner, with the NORDCAN database and webtool. Nordcan.R was also successfully used in a non-Nordic country.

## Accessibility to software and specifications

Documentation for nordcan.R, the application itself and the packages it consists of can be downloaded from the Cancer Registry of Norway GitHub repositories NORDCAN, basicepistats, nordcanepistats, nordcansurvival, nordcancore and nordcanpreprocessing (43). The code and packages are free to use as is under the given license. The NORDCAN work group and IT-group maintains and further develops nordcan.R for NORDCAN purposes and needs. Conversion tables, cancer dictionary and other documentation used in NORDCAN and nordcan.R which is not available through the GitHub-pages can be shared from the secretariat upon request. Questions can be directed to head of the NORDCAN secretariat, Siri Larønningen (sla@kreftregisteret.no). 

## Data availability statement

Publicly available datasets were analyzed in this study. This data can be found here: https://github.com/CancerRegistryOfNorway/NORDCAN, https://nordcan.iarc.fr/en.

## Author contributions

Main writers of manuscript: SL, HS, JM. Corrections, improvements and approval of final version: All authors. Developers of nordcan.R: JM, SP, HT, BA. Statistical analysis: AS, DK. All authors contributed to the article and approved the submitted version.

## References

[1] Ringertz N. Epidemiology of gastrointestinal cancers in Scandinavia. I. Report on Denmark, Finland, Norway, and Sweden. Natl Cancer Institute Monograph (1967) 25:219–39. Available at: https://europepmc.org/article/med/3465196. 6033054

[2] Hakulinen T Andersen A Malker B Pukkala E Schou G Tulinius H. Trends in cancer incidence in the Nordic countries. A collaborative study of the five Nordic Cancer Registries. Acta Pathol Microbiol Immunol Scand Suppl (1986) 288:1–151. 3465196

[3] Jensen OM. Atlas of cancer incidence in the Nordic countries: a collaborative study of the five Nordic cancer registries. Pura Musta, Helsinki: Nordic Cancer Union (1988).

[4] Tulinius H Storm H Pukkala E Andersen A Ericsson J. Cancer in the Nordic countries, 1981-86. APMIS (1992) 100(31). Acta pathologica, microbiologica et immunologica scandinavica. Supplementum. p. 194. Available at: https://pascal-francis.inist.fr/vibad/index.php?action=getRecordDetail&lang=en&idt=730737. 1301768

[5] Engeland A Haldorsen T Tulinius H Vaittinen P Tretli S Hakulinen T. Prediction of cancer incidence in the Nordic cancer up to the years 2000 and 2010. APMIS (1993) 101(38):1–124. Acta pathologica, microbiologica et immunologica scandinavica. Supplementum. Available at: https://pascal-francis.inist.fr/vibad/index.php?action=getRecordDetail&lang=en&idt=589545. 8297639

[6] Møller B Fekjær H Hakulinen T Tryggvadóttir L Storm HH Talback M. Prediction of cancer incidence in the Nordic countries up to the year 2020. Eur J Cancer Prev (2002) 11. Available at: https://www.researchgate.net/profile/Hans-Storm/publication/11025416_Prediction_of_cancer_incidence_in_the_Nordic_countries_up_to_the_year_2020/links/0046351d66737db360000000/Prediction-of-cancer-incidence-in-the-Nordic-countries-up-to-the-year-2020.pdf. 12442806

[7] Storm HH. Nordic cancer registration, a review of an invaluable source and example for surveillance, research and public health for more than 70 years. Norsk Epidemiol (2022) 30(1-2):25–30. doi: 10.5324/nje.v30i1-2.4973

[8] Bray F Parkin DM. Evaluation of data quality in the cancer registry: principles and methods. Part I: comparability, validity and timeliness. Eur J Cancer (2009) 45(5):747–55. doi: 10.1016/j.ejca.2008.11.032 19117750

[9] Parkin DM Bray F. Evaluation of data quality in the cancer registry: principles and methods Part II. Completeness. Eur J Cancer (2009) 45(5):756–64. doi: 10.1016/j.ejca.2008.11.033 19128954

[10] Larønningen S Ferlay J Beydogan H Bray F Engholm G Ervik M. NORDCAN: Cancer Incidence, Mortality, Prevalence and Survival in the Nordic Countries, Version 9.2. Oslo, Norway: Association of the Nordic Cancer Registries. Cancer Registry of Norway (2022).

[11] Engholm G Ferlay J Christensen N Bray F Gjerstorff ML Klint Å. NORDCAN–a Nordic tool for cancer information, planning, quality control and research. Acta Oncol (2010) 49(5):725–36. doi: 10.3109/02841861003782017 20491528

[12] Larønningen S Larsen IK Møller B Engholm G Storm HH Johannesen TB. NORDCAN - Cancer data from the Nordic countries. Oslo: Cancer Registry of Norway (2013).

[13] Pukkala E Engholm G Højsgaard Schmidt LK Storm H Khan S Lambe M. Nordic Cancer Registries–an overview of their procedures and data comparability. Acta Oncol (2018) 57(4):440–55. doi: 10.1080/0284186X.2017.1407039 29226751

[14] European Union. General Data Protection Regulation - GDPR (2018). Available at: https://gdpr-info.eu/.

[15] The R Foundation. The R Project for Statistical Computing (2022). Available at: https://www.r-project.org/.

[16] StataCorp. Stata (2022). Available at: https://www.stata.com/.

[17] International Agency for Research on Cancer (IARC). Check and conversion Program (2018). Available at: http://www.iacr.com.fr/index.php?option=com_content&view=article&id=72:iarccrgtools&catid=68&Itemid=445.

[18] World Health Organization. International Classification of Diseases for Oncology. 3rd ed. Malta: WHO Press, World Health Organization (2013). First Revision (ICD-O-3).

[19] World Health Organization. International Classification of Diseases (2019). Available at: https://icd.who.int/browse10/2019/en.

[20] International Agency for Research on Cancer (IARC) World Health Organization (WHO) International Association of Cancer Registries (IACR) European Network of Cancer Registries (ENCR). International rules for multiple primary cancers. (ICD-O Third Edition). Lyon: IARC (2004). Available at: http://www.iacr.com.fr/images/doc/MPrules_july2004.pdf.

[21] NORDCAN work group. Call for data - Incidence (2022). Available at: https://github.com/CancerRegistryOfNorway/NORDCAN/wiki/Call-for-data—Incidence.

[22] NORDCAN work group. Call for data - Mortality (2022). Available at: https://github.com/CancerRegistryOfNorway/NORDCAN/wiki/Call-for-data—Mortality.

[23] NORDCAN work group. Call for data - Population (2022). Available at: https://github.com/CancerRegistryOfNorway/NORDCAN/wiki/Call-for-data-Population.

[24] NORDCAN work group. Call for data - Survival (2022). Available at: https://github.com/CancerRegistryOfNorway/NORDCAN/wiki/Call-for-data-Survival.

[25] NORDCAN work group. NORDCAN - The cancer dictionary (2022). Available at: https://nordcan.iarc.fr/en/database#bloc1.

[26] NORDCAN work group. The Cancer Registry of Norway metadatabase: Entity (2022). Available at: https://metadata.kreftregisteret.no/variables/detail/814?tabIndex=4.

[27] NORDCAN work group. ICD10 - ICD6-7/ICD8/ICD9. The Cancer Registry of Norway metadatabase (2022). Available at: https://metadata.kreftregisteret.no/tables/view/7.

[28] NORDCAN work group. nordcan.R output (2022). Available at: https://github.com/CancerRegistryOfNorway/NORDCAN/wiki/nordcan.R-output.

[29] NORDCAN work group. Nordcan survival methods (2022). Available at: https://github.com/CancerRegistryOfNorway/NORDCAN/wiki/nordcansurvival.

[30] Coviello E Dickman P Seppa K Pokhrel A. STNET: Stata module to calculate net survival. Boston College Department of Economics: EconPapers (2020). Available at: https://econpapers.repec.org/software/bocbocode/S457533.htm.

[31] NORDCAN work group. Protecting confidentiality in NORDCAN (2021). Available at: https://github.com/CancerRegistryOfNorway/NORDCAN/wiki/Protecting-confidentiality-in-NORDCAN.

[32] Hundepool A Domingo-Ferrer J Franconi L Giessing S Nordholt ES Spicer K. Statistical disclosure control. Wiley New York (2012), vol 2.

[33] Policy on protecting confidentiality in tables of birth and death statistics. United Kingdom: Office for National Statistics (2018). Available at: https://www.ons.gov.uk/methodology/methodologytopicsandstatisticalconcepts/disclosurecontrol/policyonprotectingconfidentialityintablesofbirthanddeathstatistics.

[34] The Norwegian Data Protection Authority. A guide to the anonymisation of personal data. Oslo: The Norwegian Data Protection Authority (2015).

[35] NORDCAN work group. Glossary of statistical terms (2022). Available at: https://nordcan.iarc.fr/en/additional-information?tab=2.

[36] Ervik M. Global Cancer Observatory: Cancer Over Time (2021). Available at: https://gco.iarc.fr/overtime/en (Accessed 2022 20.09).

[37] Netherlands Cancer Registry and Netherlands Comprehensive Cancer Organisation (IKNL). NKR cijfers (2022). Available at: https://iknl.nl/nkr-cijfers (Accessed 2022 10.10).

[38] Cancer Registry of Norway. STINE - Cancer Registry of Norway databank (2022). Available at: https://sb.kreftregisteret.no/.

[39] International Agency for Research on Cancer (IARC). Cancer Incidence in Five Continents - Volume XII (2022). Available at: http://www.iacr.com.fr/index.php?option=com_content&view=article&id=90&Itemid=566.

[40] European Network of Cancer Registries. Call for data (2022). Available at: https://www.encr.eu/call-for-data.

[41] NORDCAN work group. Quality tables (2022). Available at: https://github.com/CancerRegistryOfNorway/NORDCAN/wiki/Specification-quality-tables.

[42] European Health Data & Evidence Network (EHDEN). EHDEN: Becoming the trusted open science community built with standardised health data via a European federated network. (2022). Available at: ehden.eu/consortium-partners/.

[43] Miettinen J Aagnes B Pejcic S Tian H. nordcan.R. (2022). Available at: https://github.com/CancerRegistryOfNorway/NORDCAN/tree/master/releases.

